# Implications of Membrane Binding by the Fe-S Cluster-Containing N-Terminal Domain in the *Drosophila* Mitochondrial Replicative DNA Helicase

**DOI:** 10.3389/fgene.2021.790521

**Published:** 2021-12-07

**Authors:** Minyoung So, Johnny Stiban, Grzegorz L. Ciesielski, Stacy L. Hovde, Laurie S. Kaguni

**Affiliations:** ^1^ Department of Biochemistry and Molecular Biology and Center for Mitochondrial Science and Medicine, Michigan State University, East Lansing, MI, United States; ^2^ Department of Biology and Biochemistry, Birzeit University, Birzeit, Palestine; ^3^ Institute of Biosciences and Medical Technology, University of Tampere, Tampere, Finland; ^4^ Department of Chemistry, Auburn University at Montgomery, Montgomery, AL, United States

**Keywords:** iron-sulfur clusters, genome stability, liposomes, membrane binding, mitochondria, replicative helicase, NUBPL/Ind1

## Abstract

Recent evidence suggests that iron-sulfur clusters (ISCs) in DNA replicative proteins sense DNA-mediated charge transfer to modulate nuclear DNA replication. In the mitochondrial DNA replisome, only the replicative DNA helicase (mtDNA helicase) from *Drosophila melanogaster (Dm)* has been shown to contain an ISC in its N-terminal, primase-like domain (NTD). In this report, we confirm the presence of the ISC and demonstrate the importance of a metal cofactor in the structural stability of the *Dm* mtDNA helicase. Further, we show that the NTD also serves a role in membrane binding. We demonstrate that the NTD binds to asolectin liposomes, which mimic phospholipid membranes, through electrostatic interactions. Notably, membrane binding is more specific with increasing cardiolipin content, which is characteristically high in the mitochondrial inner membrane (MIM). We suggest that the N-terminal domain of the mtDNA helicase interacts with the MIM to recruit mtDNA and initiate mtDNA replication. Furthermore, *Dm* NUBPL, the known ISC donor for respiratory complex I and a putative donor for *Dm* mtDNA helicase, was identified as a peripheral membrane protein that is likely to execute membrane-mediated ISC delivery to its target proteins.

## Introduction

Iron-sulfur clusters (ISCs) are ancient cofactors thought to be involved in the emergence of the origin of life (Koonin and Martin, 2005). ISCs are now found in a wide range of enzymes as prosthetic groups, and fulfill multifaceted roles in cellular metabolism (Beinert et al., 1997; Beinert and Kiley, 1999; Beinert, 2000; Stiban et al., 2016; Khodour et al., 2019). During the last decade, ISCs have been identified in diverse nucleic acid processing enzymes such as primases (Weiner et al., 2007; O’Brien et al., 2017), polymerases (Netz et al., 2012), helicases (Rudolf et al., 2006; Fan et al., 2008; Stiban et al., 2014), nucleases (Yeeles et al., 2009; Pokharel and Campbell, 2012; Sparks et al., 2012), glycosylases (Boal et al., 2009), tRNA thiolating enzymes (Romsang et al., 2018), and transcription factors (Khoroshilova et al., 1997). ISCs in DNA processing enzymes contribute primarily to structural stability, and only a few proteins such as glycosylases (Boal et al., 2009) and some DNA repair helicases (Rudolf et al., 2006) are known to contain ISCs that modulate enzymatic activity (White and Dillingham, 2012). A novel role for ISCs in DNA processing enzymes, DNA-mediated charge transfer (DNA-CT), has been demonstrated in nuclear DNA repair (in endonuclease III, MutY, DinG and XPD) and transcription (in SoxR) (Boon et al., 2003; Fuss et al., 2015; Grodick et al., 2015; Arnold et al., 2016). Though ISCs have been detected in many nuclear replicative proteins such as DNA polymerases α, δ, and ε (Netz et al., 2012), and primases (Klinge et al., 2007; Vaithiyalingam et al., 2010), a clear role for them in DNA replication had not been described until recently. DNA binding by human primase was shown to be regulated by the oxidation state of its ISC, and electron transport by DNA-CT to lead to reduction of the ISC and subsequent primase handoff, such that DNA polymerase α serves as a putative electron transfer partner at the replication fork (O’Brien et al., 2017; Tse et al., 2017). More recently however, charge transfer between [4Fe-4S] proteins and DNA was shown to be unidirectional and kinetically unfavorable in the opposite direction, such that oxidants or reductants may be necessary (Teo et al., 2019).

Mitochondria contain their own genome, and replication, repair, transcription, and translation systems, and are essential for ISC biogenesis in animal cells (Maio and Rouault, 2015). Whereas a considerable number of DNA processing proteins have been found to have ISCs in the nucleus, the presence of ISCs in mitochondrial DNA (mtDNA) metabolism has only recently been explored (Khodour et al., 2019). Dna2 helicase-nuclease (Budd et al., 2006; Pokharel and Campbell, 2012) and exonuclease 5 (Burgers et al., 2010) have been reported as ISC-containing DNA processing proteins that function in mitochondria (Stiban et al., 2016). Dna2 serves roles in maintaining both nuclear and mitochondrial genomes (Duxin et al., 2009). The ISC in Dna2 has been suggested to provide a functional link between its nuclease and helicase domains, because mutagenesis in the ISC-coordinating cysteine residues reduces both nuclease and ATPase activities (Pokharel and Campbell, 2012). In human mitochondria, Dna2 stimulates mitochondrial DNA polymerase activity, and removes RNA primers in replication, and processes 5′-flap intermediates in long-patch base excision repair (Zheng et al., 2008). Recently, it has been demonstrated that the ISC in Dna2 is required for structural stability, DNA binding, ATPase, helicase and nuclease activities (Mariotti et al., 2020). Yeast exonuclease 5, which is essential in mitochondrial DNA replication and recombination, contains conserved cysteine residues to coordinate an ISC (Burgers et al., 2010). Its human homolog has been demonstrated to contain an ISC that is critical for its nuclease activity, although human exonuclease 5 lacks a mitochondrial leader sequence (Sparks et al., 2012). Additionally, our group has shown that the mitochondrial replicative DNA helicase (mtDNA helicase) from *Drosophila melanogaster* (*Dm*) contains a 2Fe-2S ISC in its N-terminal, primase-like domain; the ISC plays roles in mtDNA binding and protein stability (Stiban et al., 2014). The homologous primase-helicase (gp4) in T7 bacteriophage coordinates a zinc ion through the homologous cysteines in its N-terminal primase zinc binding domain (Kato et al., 2003). Interestingly, human *Hs* mtDNA helicase (TWINKLE) does not bind either metal, and the cysteine residues are not conserved (Supplementary Figure S1; Kaguni and Oliveira, 2016). The human and *Drosophila* mtDNA helicases share 40% sequence identity and whereas sequence conservation is present throughout the protein, it is highest in the C-terminal helicase domain. This evolutionary change from a zinc ion to an ISC and to the absence of a metal is intriguing, because the function of the N-terminal primase-like domains of animal mtDNA helicases has not yet been established firmly.

Mitochondrial NUBPL (nucleotide-binding protein-like), also called Ind1 (ISC assembly protein known to be required specifically for NADH dehydrogenase) (Sheftel et al., 2009; Mimaki et al., 2012) in humans, transfers 4Fe-4S clusters to complex I at the terminal stage of the ISC assembly process in mitochondria (Bych et al., 2008; Sheftel et al., 2009; Lill et al., 2012). (Though both descriptors are used for the protein, we will continue using only NUBPL for simplicity). A NUBPL deletion mutant in the yeast *Yarrowia lipolytica* shows only ∼30% residual activity and ∼20% of the relative abundance of complex I compared to wild type (Sheftel et al., 2009). A knockdown mutant of NUBPL in human HeLa cells showed a 3- to 4-fold decrease in complex I activity and reduced complex I assembly (Sheftel et al., 2009). The human protein was also identified as a disease-related gene from a cohort with complex I deficiency symptoms (Calvo and Mootha, 2010). NUBPL belongs to the Mrp/MinD family in the P-loop NTPase superfamily (Leipe et al., 2002). The Mrp/MinD family encompasses eight subfamilies, with protein members that have diverse roles in all cellular organelles. These functionally different family members show structural similarity, sharing a featured KGG signature in the Walker A motif (Leipe et al., 2002). All proteins in the Mrp/MinD family are thought to be dimers because the conserved lysine residue in the signature interacts with the terminal oxygen atom of the β phosphate group of ATP that binds in the other protomer (Leipe et al., 2002; Tezcan et al., 2005). A specific membrane association is observed in some of the proteins in this family; they use an amphipathic helix either to bind to the membrane or to transport an amphipathic helix to it (Vecchiarelli et al., 2016). Association to a specific membrane is observed in some proteins in this family (Mateja et al., 2009; Mariappan et al., 2011). To date, these common features have not been evaluated in proteins of the NBP35/Mrp subfamily to which NUBPL belongs.

We report here the identification of the *Drosophila* protein encoded by the CG3262 gene as the homolog of human NUBPL. The protein was originally identified in the mitochondrial proteome as an interacting partner of the *Dm* mtDNA helicase by high-throughput coaffinity purification mass spectrometry (Guruharsha et al., 2011). We evaluate the possibility that it may serve as the ISC transfer protein for *Dm* mtDNA helicase by documenting its mitochondrial localization, cofactor-independent dimerization and membrane binding properties. Notably, we demonstrate that the profile of mtDNA replication intermediates in cells overexpressing *Dm* NUBPL bears a striking resemblance to that of elevated helicase levels, suggesting a functional relationship between the two proteins. Finally, we report membrane binding by both the *Dm* NTD and *Dm* NUBPL, and that in the presence of liposomes, FL mtDNA helicase shows enhanced ATPase activity suggesting a key role of membrane binding to genome stability. Because human NUBPL localizes to the membrane to mediate ISC transfer to its target protein, complex I of the electron transport chain (ETC), it seems likely that this also occurs in *Drosophila*. Similarly, the docking of *Dm* NUBPL and the *Dm* mtDNA helicase may be necessary for ISC transfer and the initiation of replication.

## Materials and Methods

### Cell Culture, Generation of Stable Cell Lines, and Recombinant Protein Production in *Drosophila* S2 Cells

To express a C-terminally His-tagged *Dm* NTD (amino acids Met^1^-Ala^333^), *Drosophila* S2 cells were cultured and a stable cell line was generated as described previously (Matsushima and Kaguni, 2009). The stable cell line was grown in suspension culture in Insect-XPRESS™ protein-free insect cell medium (Lonza), and protein expression was induced with 0.2 mM CuSO_4_. After 3 days of induction, cells were harvested and mitochondria were isolated by differential centrifugation. A mitochondrial extract was prepared and purified further by nickel-nitrilotriacetic acid (Ni-NTA) affinity chromatography as described earlier (Stiban et al., 2014).

### cDNA Preparation, Vector Construction, and Generation of Transient Cell Lines

The CG3292 cDNA **(**Berkeley *Drosophila* Genome Project *Drosophila* Gene Collection clone, RE72832) was purchased from the *Drosophila* Genomics Resource Center. Because the RE72832 clone lacks 16 base pairs in its ORF, the missing nucleotides (5′-GCA GTT AAT TTT GCC T-3′) were inserted by two cycles of site-directed mutagenesis using the following primers: LSK MS11-1F 5′ GGA AAA AGC ACC GTG TTT TGC CTG CAG CTT GGC AAA AC-3′, LSK MS11-1R 5′-GTT TTG CCA AGC TGC AGG CAA AAC ACG GTG CTT TTT CC-3, LSK MS11-2F 5′-GGA AAA AGC ACC GTG GCA GTT AAT TTT GCC TGC AGC-3′, LSK MS11-2R 5′-GCT GCA GGC AAA ATT AAC TGC CAC GGT GCT TTT TCC-3’. The FL ORF was confirmed by DNA sequencing and designated as pFlc-NUBPL.

The pMt-EGFP and pMt-HA vectors were constructed by insertion of PCR-amplified EGFP (enhanced GFP, 209 amino acids) or HA fragments (hemagglutinin, 9 amino acids), respectively, into the *Drosophila* expression vector, pMt (Matsushima and Kaguni, 2009). The following primers were used for the pMt-EGFP construct (LSK MS1F 5′-GGA GGA TCC ATG GTG AGC AAG GGC GAG GA-3′ and LSK MS1R 5′-TCC ACT AGT TTA CTT GTA CAG CTC GTC CAT GC-3′) and for the pMt-HA construct (LSK MS3F 5′ GGA GGA TCC TAC CCA TAC GAT GTT C-3′ and LSK MS3R 5′-AAC ACT AGT CTA CTA CAA GCT AGC-3′).

For both C-terminally EGFP-tagged *Dm* NUBPL (M^1^-H^293^) and C-terminally HA-tagged *Dm* NUBPL (M^1^-H^293^), the ORF of the FL CG3292 was amplified by PCR from pFlc-NUBPL with following primers: LSK MS13F, 5′-GGA CTC GAG ATG GAG CGT CTA TTG ATC-3′ and LSK MS13R, 5′- CCC GGA TCC ATG TGC ACT GTT ATT TTG-3.’ The PCR products were ligated to pMt-EGFP to obtain pMt-EGFP-NUBPL, and pMt-HA to obtain pMt-HA-NUBPL.

S2 cells were transiently transfected with pMt-EGFP-NUBPL and pMt-HA-NUBPL by subculture and transfection as in the generation of stable cell lines of the *Dm* NTD, except that the selection procedure was omitted. The recombinant *Dm* NUBPL proteins were induced for 3 days with 0.2 mM CuSO_4_ at 48 h after transfection.

For *E. coli* cell expression of the N-terminally His-tagged NUBPL (Met^25^-His^293^), the ORF was amplified by PCR from pFlc-NUBPL with the following primers: LSK MS25F 5′-TTA GGA TCC ATG GCG CGG GGA TTG C-3′ and LSK MS17R 5′-CCA GTC GAC CTA CTA CAA GCT AGC GTA ATC-3’. The PCR product was then ligated into the pET28a vector (Novagen), generating pET28a-NUBPL. An ISC coordinating-deficient variant (C214A/C217A) was constructed by site directed mutagenesis of pET28a-NUBPL using following primers: LSK MS19F 5′-GAG AAC ATG AAG TAC ACC ATT GCT CAG AAC GCT AAT CAA CGA TTG GAG TTT TTT AAA G-3′ and LSK MS 19R 5′-CTT TAA AAA ACT CCA ATC GTT GAT-3’.

### Overexpression and Purification of Recombinant Proteins from *E. coli*

The N-terminally His-tagged *Dm* mtDNA helicase NTD (Asn^24^-Ala^333^) and the ISC deficient variants (C68A/C71A) and (C102A/C105A) were produced by overexpression in *E. coli* BL21 cells and purified by Ni-NTA affinity chromatography as described previously (Stiban et al., 2014), except that Superdex 200 HR 10/30 (GE Healthcare) gel filtration chromatography was substituted for the glycerol gradient sedimentation.

The N-terminally His-tagged NUBPL (Met^25^-H^293^ is) and its ISC-deficient variant (C214A/C217A) were produced by overexpression in *E. coli* BL21 cells (Novagen) in auto-induction media as described (Gajewski et al., 2016). Briefly, transformed cells were cultured at 37°C with aeration until an OD_600_ of 1 was reached. Then, the cultures were shifted to 25°C until an OD_600_ of ∼15–20 was reached (∼48–60 h).

Cell harvest and protein purification were performed as described (Stiban et al., 2014). Briefly, after harvest, cells were suspended in cold Tris-sucrose buffer (50 mM Tris-HCl, pH 7.5, 10% sucrose, 250 mM NaCl, 1.5% *n*-dodecyl β-D-maltoside, 2 μg/ml of leupeptin, 1 mM phenylmethylsulfonyl fluoride, 5 mM β-mercaptoethanol, and 10 mM sodium metabisulfite) and lysed by a freeze-thaw cycle. The resulting lysate was centrifuged 17,000 × *g* for 50 min at 4°C and the recombinant *Dm* NUBPL proteins were purified by Ni-NTA affinity chromatography. Similar approaches were taken to purify FL *Dm* and human *Hs* mtDNA helicase and the RNA polymerase domain (RPD) of mtDNA helicase.

### Potassium Ferricyanide Staining

After SDS-PAGE, gels were immersed in potassium ferricyanide solution (100 mM potassium ferricyanide, 50 mM Tris-HCl, pH 7.5, 100 mM NaCl) in the dark for 1 h. The gels were transferred to freshly-prepared color-developing solution (10% methanol, 10% trichloroacetic acid) until the protein bands were visible.

### Metal Replacement Assay

To produce the apo-NTD, the protocol of Kennedy and Beinert (Kennedy and Beinert, 1988) was modified as follows. EDTA and potassium ferricyanide were added to the NTD in a 100-fold and 50-fold molar excess in 1X replacement buffer (10 mM NH_4_HCO_3_, pH 7.5, 12% glycerol). The mixtures were kept on ice for 10 min to allow oxidation and extraction of the ISC. The apo-NTD was then desalted on a Zeba™ spin desalting column (40 kDa MWCO) and the buffer was exchanged with 2X replacement buffer. Then, the UV-vis absorption spectrum of the apo-NTD was measured in buffer A (250 mM NaCl, 35 mM Tris-HCl pH 7.5, 12% glycerol, and 5 mM β-mercaptoethanol) in a Hewlett-Packard 8453 spectrophotometer with a quartz cuvette (1 cm path length). For Zn^2+^ replacement, the apo-NTD was incubated with a 50-fold molar excess of ZnSO_4_ on ice for 1 h. Unbound Zn^2+^ was removed by desalting, and the UV-vis spectrum of the protein was measured.

Because removal of the ISC led to precipitation of the protein, a modified protocol was performed to achieve ISC removal and metal replacement at the same time. The NTD was incubated in the presence of a 100-fold excess of ZnSO_4_, a 100-fold molar excess of EDTA and a 50-fold molar excess of potassium ferricyanide. The mixture was incubated on ice for 1 h. Unbound Zn^2+^ was removed by desalting and the UV-vis spectrum of the protein was measured.

### Protein Stability Assay

Purified NTD, metal-substituted NTD and purified NTD variants (C68A/C71A) and (C102A/C105A) were incubated on ice after their initial absorption spectra were taken. At the indicated times, the samples were centrifuged at 20,000 × g for 30 s to remove precipitated material and absorption spectra were measured from supernatant fractions. Protein concentration was calculated as described previously (Stiban et al., 2014).

### Inductively Coupled Plasma Optical Emission Spectrometry

After incubation of the metal-substituted NTD for 71 h on ice, protein concentration was determined by A280 measurement, and 1.5 and 0.8 nmol protein from different preparations were centrifuged for 1 h at 20,000 × g to remove protein precipitates. Both pellet and supernatant samples were boiled in the presence of 0.5 ml nitric acid (15.7 N) and 0.5 ml H_2_O_2_ (30%) until all liquid was evaporated. A control sample was prepared using 1× replacement buffer without protein. After evaporation, all samples were resuspended in 2 ml of 2% nitric acid. Fe and Zn standards were prepared using (Fe(NO_3_)_3_·9H_2_O and ZnSO_4_·7H_2_O in 2% nitric acid solutions of 50, 100, 150, 200, 300, 400, 500, 750, and 1,000 ppb of both ions. Samples and standards were analyzed in an ICP-OES Varian 710-ES Axial spectrometer (Agilent Technologies) using the ICP Expert^TM^ II software.

### Fluorescence Microscopy

Transiently-transfected, C-terminally EGFP-tagged NUBPL-expressing S2 cells and wild type S2 cells were grown to 70% confluence on a cover glass inside a six-well tissue culture plate (Corning). Mitochondria within the cells were stained with MitoTracker Red (10 μM final concentration, Molecular Probes) for 1 h in the dark before observation in an Olympus FluoView FV1000 confocal laser scanning microscope. The instrument was equipped with 488 nm argon and 543 nm helium-neon lasers for GFP and MitoTracker Red excitation, respectively. Fluorescence emission detected with a 505–525 nm band-pass filter for GFP, and 560 nm long-pass filter for MitoTracker Red. Images were obtained using a 60X PlanApo oil objectives.

### Gel Filtration

*Dm* NUBPL (0.5 mg/ml, total 1 mg) was loaded onto a Superdex 75 HR 10/30 gel filtration column (GE healthcare) and chromatographed in gel filtration buffer (35 mM Tris-HCl pH 7.5, 350 mM NaCl, 10% glycerol, 5 mM β-mercaptoethanol) in the presence or absence of EDTA. Target protein eluted in the peak fractions was analyzed by 12% SDS-PAGE. Band intensities were measured using the ImageQuant 5.2 software after Coomassie blue staining.

### Vesicle Cosedimentation Assay

Asolectin (Sigma) in the presence or absence of additional lipids (cholesterol (Sigma) or cardiolipin (Sigma)) were dissolved in chloroform (Baker) and dried under nitrogen gas. The residual chloroform was eliminated by vacuum desiccation for 8 h. In the preparation of multilamellar liposomes, the dry lipids were hydrated for 30 min in hydration buffer (10 mM NH_4_HCO_3_, pH 7.4, 0.1 mM EDTA). The solution was mixed by vortexing until visible clots disappeared. Then, four cycles of freezing, thawing, sonication, and vortexing were performed. Large unilamellar liposomes were prepared by extrusion through a polycarbonate membrane (100 nm pore size) using a mini-extruder (Avanti Polar Lipids). After incubation of the protein and liposomes for 15 min under the stated conditions, the reaction mixtures were centrifuged at 47,000 × g in an S120-AT3 rotor (Thermo Scientific) for 1 h. Equal portions of supernatant and pellet were analyzed by 12% SDS-PAGE followed by immunoblotting or silver staining. The intensities of the protein signals on the membrane were quantified using the ImageQuant 5.2 software. The fraction of bound protein was determined dividing the band intensity of the pellet (bound protein) by those of the pellet and the supernatant together (total protein).

### Liposome Fluorescence Quenching Assay

Unilamellar liposomes containing asolectin and 0.5% BODIPY-TMR-PI(4,5)P_2_ (mol%) were prepared as previously stated. Liposomes were hydrated in LH2 buffer. The fluorescence emission spectrum of a 10 μM liposome suspension was measured at 574 nm following an excitation at 542 nm. Following sequential additions of purified NTD, the fluorescence spectra were measured. NTD was added from stock of >50 μM. Buffer controls were done by adding the same amount of buffer to the cuvette. Fluorescence measurements were performed in PTI QW4 spectrofluorimeter equipped with a temperature-controlled cuvette holder kept at 20°C.

### Full-Length *Hs* Helicase Binding to Liposomes

Both purified FL *Hs* mtDNA helicase and the RPD were used to test liposome binding using BODIPY-TMR-PI(4,5)P_2_ quenching and cosedimentation assays. Fluorescence quenching experiments were performed in a similar manner as presented earlier. Cosedimentation was also repeated using 16% CL-asolectin liposomes and 8 μg of protein.

### Malachite Green ATPase Assay

In order to measure the activity of the FL *Hs* mtDNA helicase, an ATPase/GTPase activity assay (Sigma cat. # MAK113) was performed according to the manufacturer’s instructions. In triplicate wells of a 96-well flat bottom plate, FL *Hs* helicase was incubated without or with increasing 16% CL-asolectin liposome concentrations and the volume was adjusted to 10 μl with assay buffer (40 mM Tris, 80 mM NaCl, 8 mM Mg acetate, 1 mM EDTA, pH 7.5). Background control was 10 μl of H_2_O. Phosphate standards were also prepared in triplicate wells. Master reaction mix was prepared by mixing 20 μl of assay buffer with 10 μl of 4 mM ATP per well. 30 μl of the reaction mix was added to each well, and the plate was incubated for 30 min at room temperature. To each well, 150 μl of Reagent and 50 μl H_2_O were added, and the plate was further incubated at room temperature for 30 min, followed by absorbance measurement at 650 nm using a SpectroMax M2/M2^e^ multi-well spectrophotometric plate reader, and data analysis by SoftMax Pro 53 software.

Calf thymus DNA was used to stimulate FL *Hs* helicase activity in an independent experiment following the same protocol, but with the addition of increasing amounts of DNA. Similarly, as a positive control, the ATPase activity of ΔN-helicase, termed P66 in Ziebarth et al. (2007), was measured in separate experiments.

### Protein Modeling and Multiple Sequence Alignment

To obtain a homology model of *Dm* NUBPL, the *Dm* NUBPL sequence was submitted to Phyre2 using the default parameters (Kelley et al., 2015). The dimer structure was derived by Dali (Holm and Rosenström, 2010) using the superposition of the NUBPL monomer model on the dimer crystal structure of nucleotide-binding protein AF2269 from *A. fulgidus* as a template (PDB# 3KB1). The electrostatic surface potential map of *Dm* NUBPL was generated using the APBS Tools in PyMol with the default parameters. *Dm* NUBPL was also modelled using AlphaFold software (Jumper et al., 2021).

To generate helical wheel projections, predicted secondary structures were obtained first using PsiPred (Buchan et al., 2013) from the primary amino acid sequences of the proteins. The corresponding sequence of the last predicted helix at the C-terminus of each protein was submitted to NetWheels (http://lbqp.unb.br/NetWheels/) to obtain a helical wheel projection.

In order to align human and *Drosophila* mtDNA helicases, the protein sequences (PDB accession numbers: Q96RR1 (*Hs*) and AAF52820 (*Dm*)) were subjected to BLAST analysis using the BLAST and Multiple Sequence Alignment tools on NCBI website (https://blast.ncbi.nlm.nih.gov/Blast.cgi).

### Two-Dimensional Agarose Gel Electrophoresis

The 2DAGE experiments were performed as described previously (Ciesielski et al., 2018), using mitochondrial nucleic acids (mtNA) isolated from *Drosophila* S2 cells transfected transiently with pMt-HA-NUBPL, with or without the CuSO_4_ induction. mtNA were treated with ClaI restriction endonuclease, and DNA replication intermediates (RI) were detected using the radiolabeled probe 6 that hybridizes to nts 6801–7378.

## Results

### The N-Terminal Domain of the Mature *Drosophila melanogaster* Mitochondrial DNA helicase Overexpressed in *Drosophila* S2 Cells Contains an Iron-Sulfur Cluster

**FIGURE 1:**
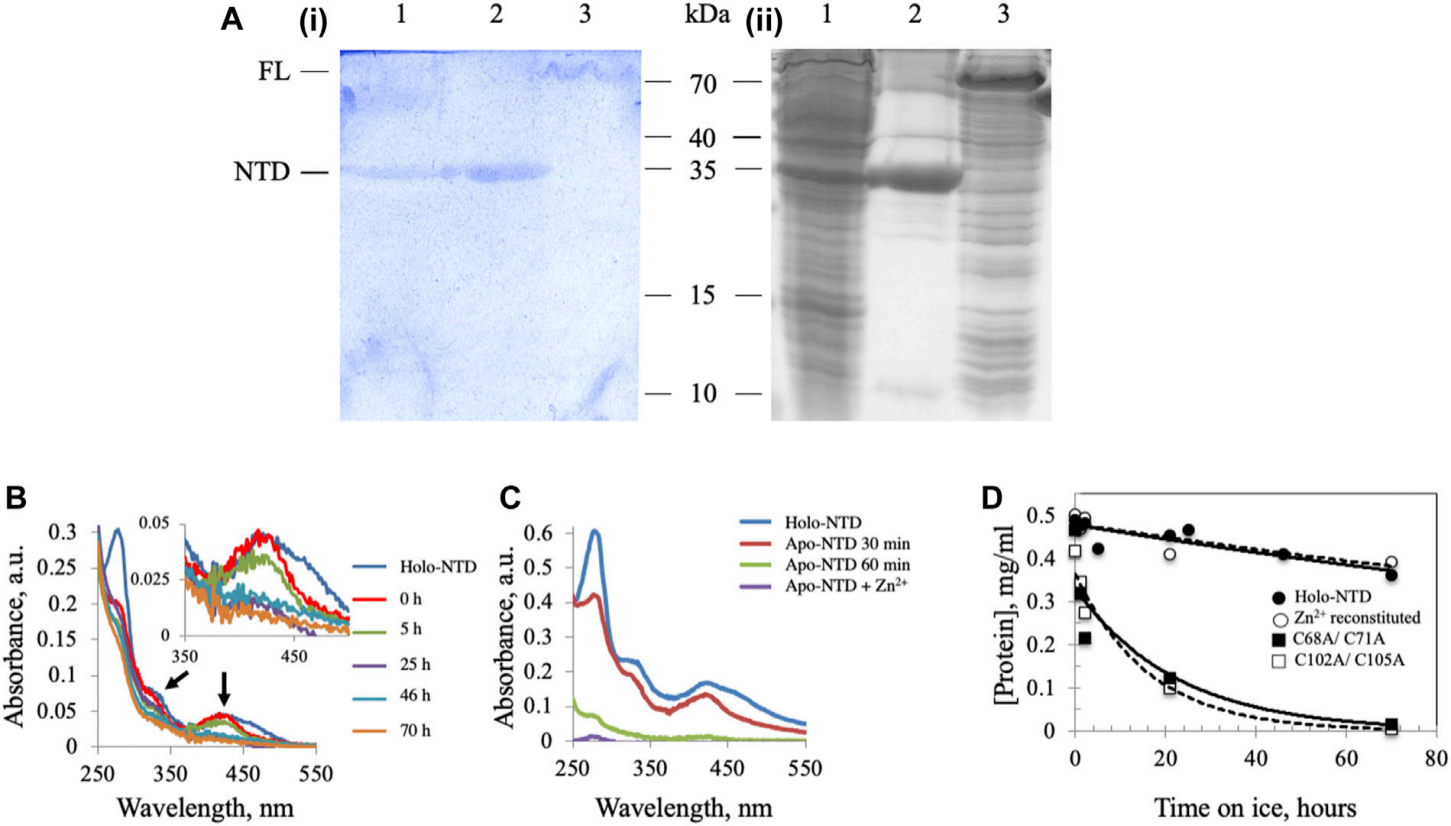
*Dm* NTD contains non-heme iron that is required for its stability *in vitro*. **(A)** Potassium ferricyanide **(i)** and Coomassie brilliant blue staining **(ii)** of partially-purified NTD overexpressed in *Drosophila* S2 cells (lane 1), purified NTD overexpressed in *E. coli* BL21 (lane 2) and overexpressed full-length *Dm* mtDNA helicase in an *E. coli* BL21 cell lysate (lane 3). **(B)** Representative absorbance spectra of wild-type and Zn^2+^-substituted NTD show a decrease of the characteristic ISC peaks (arrows and inset) with time of incubation on ice. **(C)** Extraction of the ISC from *Dm* NTD leads to protein loss. Representative absorbance spectra of native and ISC-depleted apo-NTD (30 min and 1 h post-extraction of the ISC) show decreased absorption at A280. Supplementation with Zn^2+^ post-extraction did not restore solubility of the apo-NTD. **(D)** The stability of proteins was inferred from their concentration over time. The protein concentration was calculated from the absorbance at 280 nm at the indicated time intervals. Wild-type NTD (closed circles) and Zn^2+^-substituted NTD (open circles) remain stable on ice during the experimental period, whereas the ISC-deficient variants are not (closed and open squares).

We demonstrated previously the presence of an ISC in *Dm* mtDNA helicase (Stiban et al., 2014), though the possibility remained that cluster insertion might be an artefact of the *E. coli* overexpression system. To verify the presence of an ISC in *Dm* mtDNA helicase, a recombinant form of the NTD was extracted from mitochondria isolated from *Drosophila* S2 cells and purified by Ni-NTA affinity chromatography. The partially-purified, NTD-containing fraction was separated by 15% SDS-PAGE and stained with potassium ferricyanide to detect non-heme iron ions such as those in an ISC. In this protocol, protein-bound ferrous iron reacts with potassium ferricyanide to form a royal blue complex, for which the intensity is directly proportional to the amount of iron present in the gel (Leong et al., 1992). The NTD proteins from S2 cells (Figure 1A, lane 1) and from *E. coli* BL21 cells (Figure 1A, lane 2) were detected by both Coomassie (ii) and potassium ferricyanide (i) staining. A recombinant FL *Dm* mtDNA helicase from an *E. coli* BL21 cell lysate was also detected at a position corresponding to ∼70 kDa by both staining procedures (Figure 1A, lane 3). These data indicate that the ISC is a *bona fide* component of the *Dm* mtDNA helicase.

### Binding of a Metal Cofactor Stabilizes the N-Terminal Domain of the *Dm* mtDNA Helicase

We showed previously that double-substitution variants that lack the ability to bind an ISC (C68A/C71A and C102A/C105A) are unstable and precipitate during purification (Stiban et al., 2014). To examine further the role of the ISC in protein stability, a metal replacement strategy was pursued (Kennedy and Beinert, 1988). The ISC was fully oxidized and removed by incubating the holo-NTD with excess EDTA and potassium ferricyanide, as shown by a time-dependent decrease in the characteristic ISC peaks at 325 and 420 nm upon UV-visible spectroscopy (Figure 1B). The apo-protein however, was destabilized and precipitated (Figure 1C). We then evaluated Zn^2+^ substitution before and after ISC removal. While it had no effect after ISC removal (Figure 1C), the addition of Zn^2+^ with potassium ferricyanide to achieve ISC removal and replacement at the same time resulted in the disappearance of the characteristic peaks of the ISC in the absorbance spectrum (Figure 1B). The appearance of 0.77 ± 0.1 zinc molecule per NTD was detected by Inductively Coupled Plasma Optical Emission Spectrometry (Table 1). The effect of replacing the ISC with Zn^2+^ was probed by examining protein stability over time of incubation at 0°C. The Zn^2+^-substituted NTD showed a decay curve that was nearly identical to that of the ISC-containing wild-type NTD. By comparison, the ISC binding-deficient variants showed a rapid decay (Figure 1D).

**TABLE 1:** Inductively Coupled Plasma Optical Emission Spectrometry of the *Dm* NTD after metal extraction and substitution confirms the absence of iron, and the presence of zinc in two independent preparations.

Sample	Amount (nmol)	Fe 234 (μg/ml)	Fe 259 (μg/ml)	Zn 202 (μg/ml)	Zn 206 (μg/ml)	Molar ratio (Zn:NTD)
Buffer	—	N.D.	N.D.	N.D.	N.D.	—
NTD	1.5	N.D.	N.D.	42.74	43.64	0.88 ± 0.013
NTD	0.8	N.D.	N.D.	17.73	18.74	0.69 ± 0.027

N.D., not detected.

### The N-Terminal Domain of the *Dm* mtDNA Helicase Binds to Phospholipid Membranes

**FIGURE 2:**
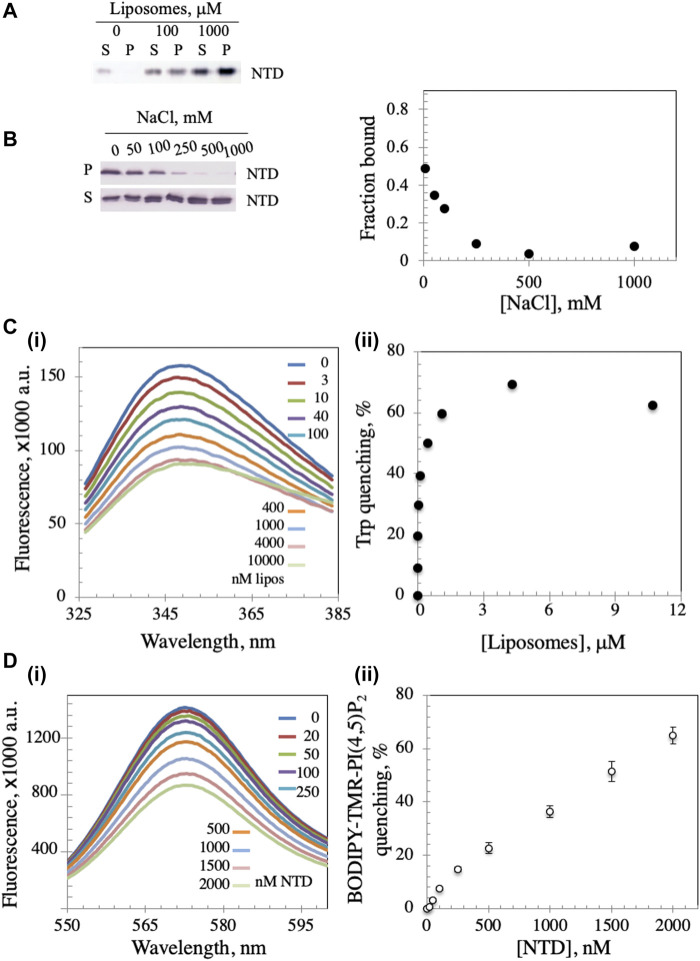
*Dm* NTD exhibit membrane binding properties. **(A)** In the liposome cosedimentation assay, 0.4 μM NTD was mixed with increasing liposome amounts and sedimented by ultracentrifugation (see *Materials and Methods*). The supernatant (S) and pellet (P) fractions were separated on 12% polyacrylamide gels and visualized by silver staining. **(B)** Increasing amount of NaCl disrupts liposome-NTD interactions. **(C)** Tryptophan fluorescence emission spectra of NTD were measured after sequential addition of different amounts of liposomes **(i)**. The observed quenching is indicative of liposome binding to NTD. Quantitative representation of the quenching is presented in **(ii)**. **(D)** The fluorescence spectra of asolectin liposomes containing 0.5% BODIPY-TMR-PI(4,5)P_2_ were measured with increasing amounts of NTD **(i)**. In **(ii)**, the percent quenching is plotted versus protein concentration. The results represent the average and standard deviation of 3 independent experiments.

The mtDNA helicase is an integral part of the minimal mitochondrial replisome, and initiation of mtDNA replication has been suggested to involve attachment of the DNA-protein apparatus to the mitochondrial inner membrane (MIM) (Rajala et al., 2014; Gerhold et al., 2015). We evaluated the possibility of membrane binding by mixing *Dm* NTD with unilamellar soybean asolectin liposomes and subjecting them to lipid vesicle cosedimentation to demonstrate protein-liposome complexes. Asolectin is a mixture of phospholipids extracted from soybean containing ∼45% phosphatidylcholine, 22% phosphatidylethanolamine, 18% phosphatidylinositol and 7% phosphatidic acid that mimics closely the composition of biological membranes. It is used widely in model membrane preparation and in liposome generation for sedimentation and membrane permeability assays (Nomura and Kurihara, 1987; Stiban et al., 2006; Avanti-Polar-Lipids, 2021; Sigma-Aldrich, 2021). We observed that in the absence of liposomes, *Dm* NTD was predominantly in the supernatant fraction (S) after ultracentrifugation, whereas in their presence, the protein was found in the pellet (P) (Figure 2A), indicating that the *Dm* NTD binds to liposomes. Liposome binding was inhibited with increasing NaCl concentrations (Figure 2B). The loss of cosedimentation of *Dm* NTD and liposomes by increased salt concentration suggests that the interaction with asolectin liposomes is electrostatic.

In order to confirm binding, intrinsic tryptophan fluorescence was measured. *Dm* NTD contains three tryptophan residues, which exhibit intrinsic fluorescence. The quantum yield of the fluorescence increases when the tryptophan residues are subjected to a hydrophobic environment, whereas it decreases in hydrophilic conditions (Kraft et al., 2009). Upon incubation of NTD with increasing liposome concentrations, we found that the quantum yield of its tryptophan fluorescence decreased in a dose-dependent manner, indicating binding to liposomes and exposure of the residues to a hydrophilic environment (Figure 2C), whereas the addition of buffer alone had no effect on tryptophan fluorescence (data not shown). Quenching was reversed by the addition of NaCl indicating salt-dependent binding (data not shown). Additionally, using fluorescently-labelled liposomes, the NTD was shown to induce the rearrangement and sequestration of BODIPY-TMR-PI(4,5)P_2_ lipids in asolectin liposomes in a dose-dependent manner (Figure 2D). Upon sequestration, BODIPY-TMR-PI(4,5)P_2_ monomers self-quench their fluorescent signal. Thus, NTD binding to liposomes was confirmed by three methods.

### The N-Terminal Domain of the *Dm* mtDNA Helicase Binds More Efficiently to Cardiolipin-Containing Liposomes

**FIGURE 3:**
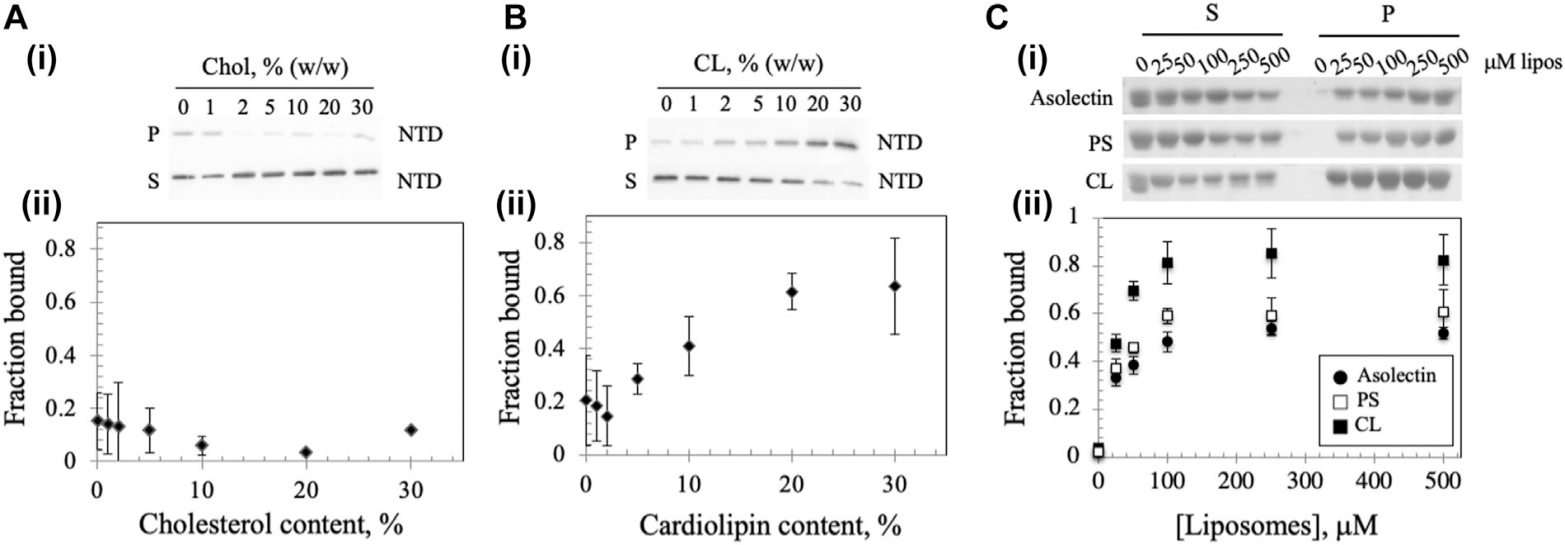
Membrane composition affects *Dm* NTD liposome binding. Binding of *Dm* NTD to liposomes that contain **(A)** cholesterol or **(B)** CL as assessed by cosedimentation are displayed in a representative gel image **(i)** and a graph of fraction bound **(ii)**. Calculation of fraction bound is described in the *Materials and Methods*. Each data point represents the mean of triplicate measurements. The error bars indicate ± standard deviation. **(C)** Liposomes containing CL and not PS show enhanced *Dm* NTD binding by cosedimentation. Varying concentrations of 15% CL-asolectin liposomes, 30% PS-asolectin liposomes or asolectin liposomes were incubated with 10 μM *Dm* NTD and separated by ultracentrifugation. The fraction bound was calculated and plotted. Each point represents the average and standard error of three different experiments.

A recent report showed that mtDNA replicative proteins in a mitochondrial lysate of human HEK293 cells cosedimented with cholesterol in flotation gradients, suggesting that the replisome binds a cholesterol-rich membrane (Gerhold et al., 2015). To investigate whether the *Dm* NTD binds to cholesterol in cholesterol-containing liposomes, we diluted standard asolectin-only liposomes with those containing cholesterol to generate variable concentrations of cholesterol-containing liposomes. The increase in cholesterol concentration had no significant effect on the bound protein fraction (Figure 3A), indicating that *Dm* NTD does not bind to cholesterol in the asolectin liposomes. In contrast, when cardiolipin (CL), an abundant lipid in the MIM (Horvath and Daum, 2013; Rappocciolo and Stiban, 2019), was added to standard asolectin liposomes, the fraction of bound *Dm* NTD increased in a CL concentration-dependent fashion: binding increased from 0.1 to 0.6 as CL concentration increased from 2 to 20% w/w, and it was saturated when liposomes contained more than 20% w/w CL (Figure 3B). This data suggests that the *Dm* NTD binds specifically to MIM as a consequence of their CL content. The results also argue that cholesterol is not involved in membrane attachment of the *Dm* NTD. In order to confirm the specificity of CL binding, liposomes were enriched with 30% phosphatidylserine (PS) the cosedimentation analysis was repeated. CL has two negative charges while PS has one, so to control for the charge effect of CL, asolectin liposomes were supplemented with 15% CL or 30% PS prior to *Dm* NTD binding. We found that enrichment of liposomes with CL enhanced markedly the binding efficiency of *Dm* NTD as compared to PS-enriched or unenriched asolectin liposomes (Figure 3C), indicating that the protein may interact with CL microdomains that are elevated in the inner membrane.

### ATPase Activity of the *Hs* Replicative mtDNA Helicase is Enhanced by Binding to Liposomes

**FIGURE 4:**
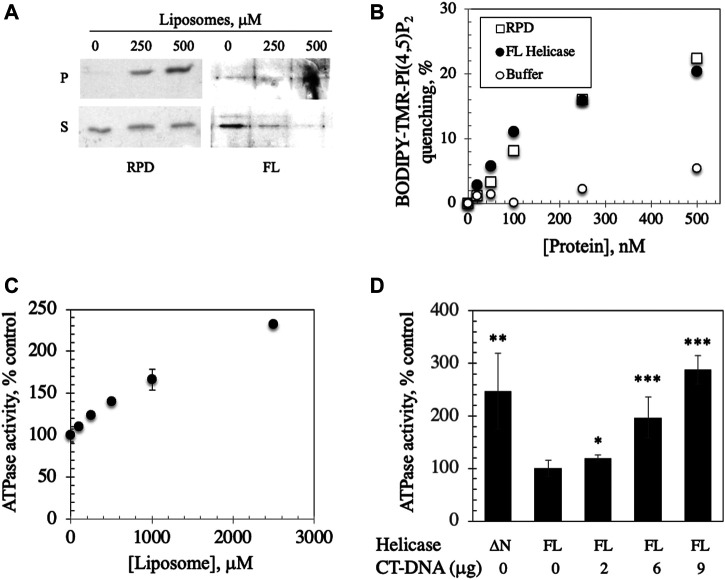
FL helicase binds to membranes and Ipid binding enhances its ATPase activity. **(A)** RPD and FL helicase cosediment with increasing liposome concentrations **(B)** Both RPD (open squares) and FL helicase (closed circles) exhibit increased liposome fluorescence quenching in a dose-dependent manner. Buffer controls (open circles) are shown. **(C)** ATPase activity of the human replicative mtDNA helicase is enhanced by binding to liposomes. Malachite green ATPase/GTPase assay was performed after incubation of 2 μg of FL *Hs* helicase with increasing CL-asolectin liposome concentrations for 30 min at room temperature. **(D)** ATPase activity of the FL helicase is DNA dependent; CT-DNA, calf thymus DNA. A variant lacking the N-terminus (ΔN) demonstrates increased activity. The error bars represent the standard deviation of three independent experiments. *, p < 0.05; **, p < 0.01; ***, p < 0.001 as compared to ATPase activity in the absence of DNA.

The physiological relevance of liposome binding to the *Dm* NTD is unclear because it has no identified activity, and we have been unable to produce a stable recombinant form of purified FL *Dm* mtDNA helicase. Thus, we evaluated lipid binding by the human FL homolog. We observed that both the FL *Hs* mtDNA helicase and its RPD domain (the RNA polymerase-like subdomain of the NTD) bound to CL-asolectin liposomes in a dose-dependent manner in the vesicle cosedimentation assays (Figure 4A). Moreover, both proteins caused quenching of BODIPY-TMR-PI(4,5)P_2_ fluorescence to the same extent (Figure 4B) arguing that the RPD subdomain of the NTD is the site of attachment to the bilayer.

We investigated the implications of binding by assessing the ATPase activity that was previously documented in the human FL helicase (Ziebarth et al., 2007) in the presence of increasing liposome concentrations. We observed that ATPase activity was stimulated by liposome binding (Figure 4C), suggesting a potentially important role for membrane binding to enhance the activity of the helicase in initiation of mtDNA replication. To confirm that the ATPase assay was indicative of helicase activity, ATP hydrolysis of FL helicase was increased with added calf thymus DNA in a dose dependent fashion (Figure 4D). A variant of helicase lacking the N-terminus (ΔN) was used as a positive control because it exhibits heightened ATPase activity (Ziebarth et al., 2007).

### Identification of Homodimeric Mitochondrial *Dm* NUBPL as a Putative Iron-Sulfur Cluster Transfer Protein for *Dm* mtDNA Helicase

Proteins containing ISCs require ISC transfer partners to insert the cluster. ISC biogenesis can occur through the cytosolic ISC assembly (CIA) or the mitochondrial ISC assembly pathways (Lill et al., 2012; Braymer and Lill, 2017). Interestingly, in the *D. melanogaster* proteome, an interaction between the protein CG3262 and the mtDNA helicase was found by high-throughput co-affinity purification coupled with mass spectrometry (Guruharsha et al., 2011). The CG3262 protein shows homology to human NUBPL, with a 44% amino acid sequence identity predicted by BLAST, and a 78.1% sequence identity to *Drosophila rhopaloa* NUBPL. NUBPL in humans and yeast is an ISC transfer protein known to be required specifically for ISC insertion into mitochondrial NADH dehydrogenase (complex I) (Bych et al., 2008; Sheftel et al., 2009; Lill et al., 2012).

**FIGURE 5:**
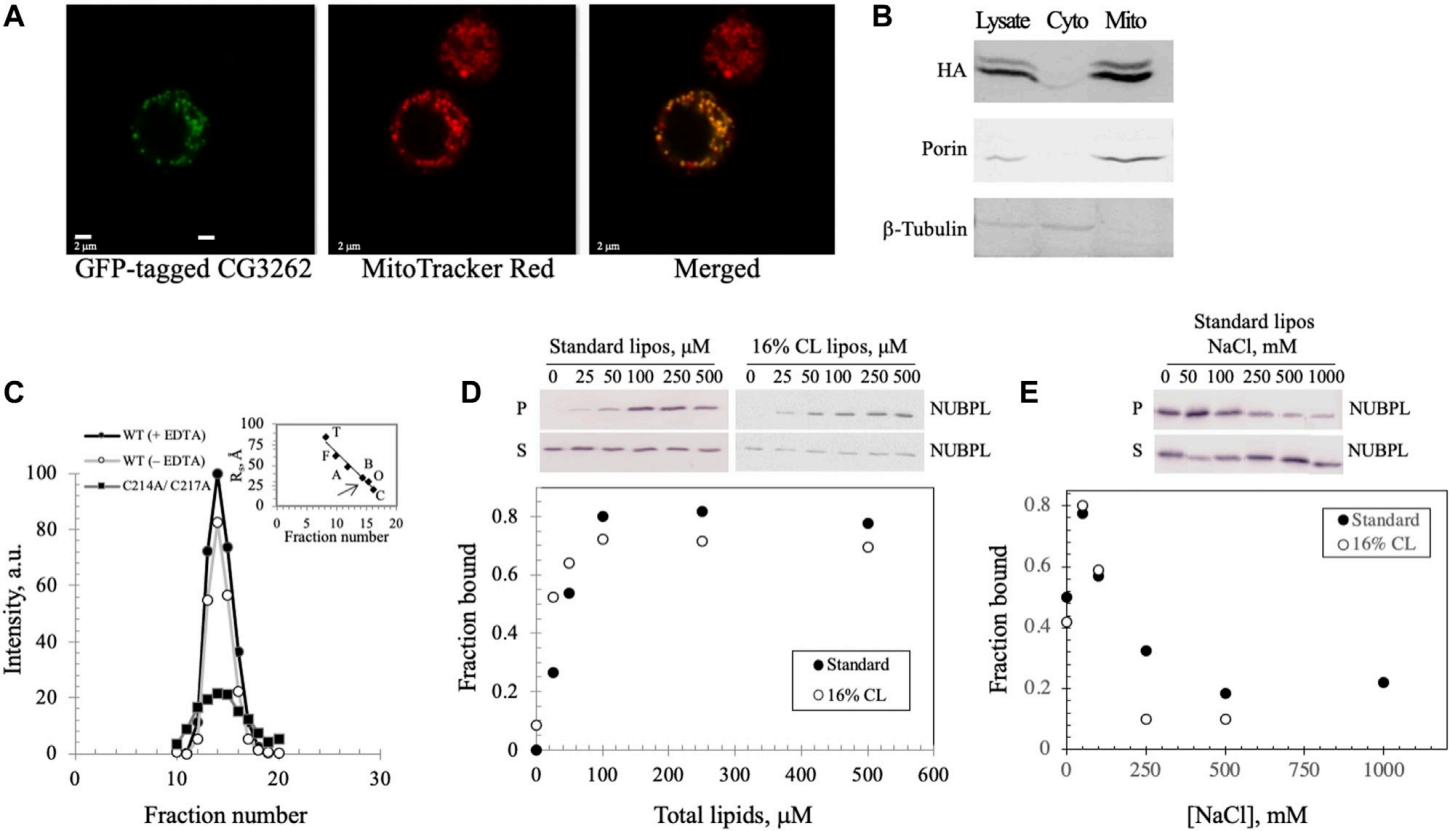
*Dm* NUBPL is a peripheral membrane protein that localizes to mitochondria and forms a homodimer regardless of the presence of an apparent ISC. **(A)** Representative confocal microscopic images of a GFP-tagged *Dm* NUBPL and **(B)** immunoblot of a HA-tagged *Dm* NUBPL show mitochondrial localization of *Dm* NUBPL. Porin and β-tubulin were used as loading controls for mitochondria and cytosol, respectively. **(C)** Gel filtration of an N-terminally His-tagged recombinant *Dm* NUBPL shows that *Dm* NUBPL in the presence (closed circles) or absence of EDTA (open circles), and an ISC coordination-deficient variant (C214A/C217A) (closed squares) produced a single peak with the same Stokes radius of 37 Å. Protein standards used for column calibration were: carbonic anhydrase (C, 20 Å, 29 kDa), ovalbumin (O, 30 Å, 45 kDa), bovine serum albumin (B, 35 Å, 66 kDa), aldolase (A, 48 Å, 158 kDa), ferritin (F, 61 Å, 440 kDa), and thyroglobulin (T, 85 Å, 669 kDa). The position of the eluted recombinant *Dm* NUBPL peak is indicated by an arrow on the standard protein curve in the inset. **(D)** Liposome cosedimentation assay shows that purified *Dm* NUBPL (0.4 μM) binds to asolectin liposomes excluding (closed circles) or including 16% w/w CL (open circles) similarly. P is the pellet fraction and S is the supernatant fraction after ultracentrifugation. **(E)** NaCl disrupts the electrostatic interactions that hold *Dm* NUBPL to liposomes in a dose-dependent manner.

To investigate the possibility that the CG3262 protein (herein termed *Dm* NUBPL) serves as the transfer protein for *Dm* mtDNA helicase, we first examined its mitochondrial localization. Primary sequence analyses with MitoProt II (Ison et al., 2016) and iPSORT (Bannai et al., 2002) predicted its mitochondrial localization with a high probability (0.7036 and positive, respectively) via a canonical N-terminal mitochondrial targeting sequence. To demonstrate mitochondrial localization *in vivo*, a C-terminal EGFP tag was inserted into the ORF containing CG3262, and the recombinant protein expressed in *Drosophila* S2 cells was evaluated by confocal microscopy (Figure 5A). The green fluorescent signal in successfully transfected cells overlapped completely the red fluorescent signal from mitochondria stained with MitoTracker Red. Moreover, subcellular fractionation of a C-terminally HA-tagged *Dm* NUBPL showed localization to the mitochondrial fraction (M) but not the cytosolic fraction (C) when proteins derived from equal cell equivalents were analyzed by 12% SDS-PAGE and immunoblotting (Figure 5B). In composite, these *in silico*, *in vivo* and *in vitro* experiments demonstrate that *Dm* NUBPL is a mitochondrial protein.

The *Dm* NUBPL monomer contains only a single CXXC motif, a common cysteine-containing motif for coordinating metal ions (Supplementary Figure S2A). Homodimerization of *Dm* NUBPL was expected because four cysteines are required typically to coordinate an ISC (Supplementary Figure S2B; Maio and Rouault, 2016). We investigated the oligomeric state of *Dm* NUBPL by gel filtration of a N-terminally His-tagged recombinant form in the absence of cofactors (Figure 5C), and in the presence of the metal chelator EDTA to reduce the possibility that it would contain an ISC or a zinc ion. We found that *Dm* NUBPL both in the presence or absence of EDTA produced a single chromatographic peak with a Stokes radius of 37 Å and an estimated molecular mass of 66 kDa, corresponding to the theoretical molecular mass of a dimer (62 kDa). This data suggests that the presence/insertion of an ISC in *Dm* NUBPL is not obligatory for its dimerization. Furthermore, we produced a *Dm* NUBPL variant lacking the ISC-coordinating capability by alanine substitution of the two cysteines in the CXXC motif. The C214A/C217A variant also demonstrated a single peak in the same fractions as the dimer-form, wild-type *Dm* NUBPL, confirming that an ISC or a metal ion is not required for dimerization. Instead, the *Dm* NUBPL dimer may form disulfide bridges in the absence of binding its metal cofactor in the presence of EDTA. We observed that the C214A/C217A variant had significantly lower solubility than the wild-type form, suggesting that as with the *Dm* NTD, the coordinating cysteines are likely important for protein stability.

### *Drosophila melanogaster* NUBPL Domain Binds to Phospholipid Membranes

Because NUBPL is an ISC transferring protein specific for respiratory complex I, it is expected to associate with the MIM where complex I resides. We investigated membrane binding by *Dm* NUBPL by mixing the purified protein with unilamellar soybean asolectin liposomes, and protein-liposome complexes were evaluated by vesicle cosedimentation. We found that in the absence of liposomes, *Dm* NUBPL was predominantly in the supernatant fraction (S) after ultracentrifugation whereas in their presence, it was found in the pellet (P) (Figure 5D, closed circles), indicating that *Dm* NUBPL binds to liposomes. The fraction of bound *Dm* NUBPL (0.8) was saturated at a concentration of 100 μM of liposomes. In contrast to *Dm* NTD liposome binding, *Dm* NUBPL binds to standard or CL-containing liposomes similarly (Figure 5D, open circles), and more tightly than *Dm* NTD, with a K_*d*_ of ∼50 μM for *Dm* NUBPL as compared to ∼100 μM for *Dm* NTD. Interestingly, we found that the bound fraction of *Dm* NUBPL at equilibration was four times higher than that of *Dm* NTD, and binding increased when the NaCl concentration was increased up to 50 mM, but it was inhibited at higher concentrations (Figure 5E). As for *Dm* NTD, the loss of cosedimentation of *Dm* NUBPL and liposomes by increased salt concentration indicates that its interaction with asolectin liposomes is electrostatic. However, we observed a residual fraction of bound *Dm* NUBPL remaining at elevated salt concentration (∼0.2), suggesting an additional type of interaction. Hydrophobic interaction seems a likely possibility because a C-terminal amphipathic helix is predicted in human, *Drosophila* and *Y. lipolytica* NUBPL by the helical wheel projection program NetWheels (Supplementary Figure S2C). Our data suggest that *Dm* NUBPL is a peripheral membrane protein, and its membrane binding likely facilitates access to its membrane-bound ISC recipient, complex I.

### *Drosophila melanogaster* NUBPL Alleviates Pausing in mtDNA Replication, and may be Related Functionally with the Activity of *Dm* mtDNA Helicase

**FIGURE 6:**
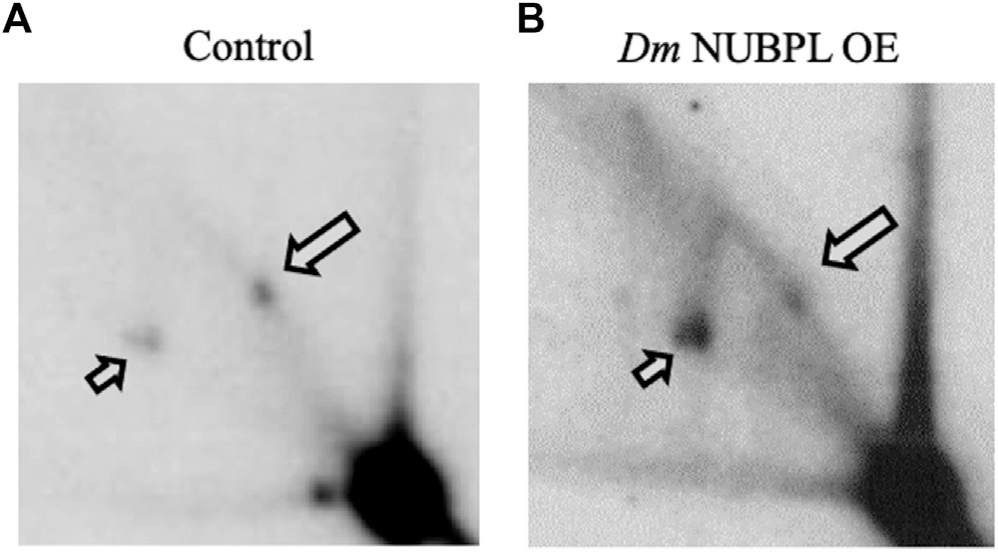
Overexpression of NUBPL alleviates mtDNA replication pausing. 2DAGE of ClaI-treated mtNA isolated from control S2 cells **(i)** or HA-NUBPL-overexpressing S2 cells **(ii)** hybridized with probe 6 (see *Materials and Methods* for details). Bold arrows indicate discrete spots on Y arcs that represent the major replication pause site at slow zone 2 (see also (Joers and Jacobs, 2013; Ciesielski et al., 2018)). Thin arrows indicate fully replicated fragment (2n).

To investigate a possible functional relationship between *Dm* NUBPL and the *Dm* replicative mtDNA helicase, we evaluated the effect of *Dm* NUBPL overexpression on mtDNA replication in S2 cells, by two-dimensional agarose gel electrophoresis. To do so, total mitochondrial nucleic acids (mtNA) obtained from sucrose gradient-purified mitochondria of the control and HA-NUBPL -overexpressing cells were cleaved by ClaI restriction endonuclease, and the resulting fragment bearing the previously identified replication pause site that defines replication slow zone 2 (Joers and Jacobs, 2013) was probed by hybridization with a homologous radiolabeled fragment. We observed that the signal from the prominent replication pause site was reduced substantially in samples from cells overexpressing *Dm* NUBPL (Figure 6), a result that is strikingly similar to the effect of the helicase overexpression in S2 cells that we have reported previously (Ciesielski et al., 2018).

**FIGURE 7:**
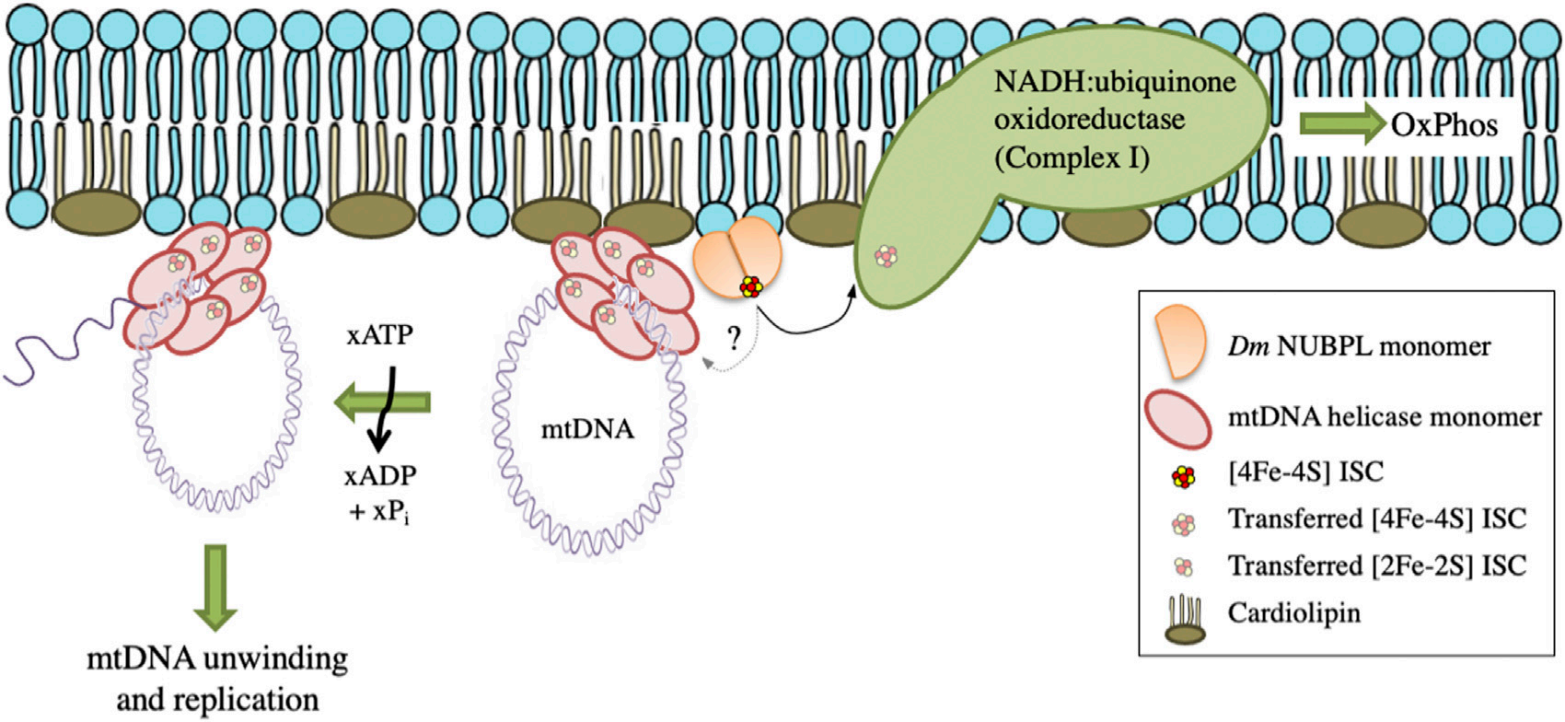
A proposed model for the transfer and importance of ISC in genome stability. *Dm* NUBPL is a peripheral mitochondrial inner membrane protein responsible for the transfer of [4Fe-4S] ISC to NADH:coenzyme Q oxidoreductase (complex I) of the mitochondrial respiratory chain. The transfer of ISC is required for an efficient oxidative phosphorylation and energy production. Similarly, *Dm* NUBPL docks to the membrane and transfers an ISC to the N-terminal domain of the mitochondrial helicase which stabilizes the protein and enhances its ATPase activity thus contributing to enhanced mtDNA replication.

These data suggest a functional interaction between *Dm* NUBPL and mtDNA helicase that is relevant to the efficiency of mtDNA replication. We propose that in the environment of the MIM, NUBPL provides mtDNA helicase with its ISC, enhancing its stability and activity, and in turn regulates the mtDNA replication process (Figure 7).

## Discussion

ISCs play significant roles in a multitude of cellular processes (Beinert et al., 1997; Beinert and Kiley, 1999; Beinert, 2000), including nucleic acid metabolism (Khodour et al., 2019). Numerous nucleic acid metabolizing enzymes contain ISCs, including the catalytic subunit of the RNA-dependent RNA polymerase responsible for the replication and transcription of severe acute respiratory syndrome coronavirus 2, in which two regions that are essential for replication and binding to viral helicase were modelled as zinc centers in the cryo-EM structure of the protein (Maio et al., 2021). Modulation of ISCs in nucleic acid metabolizing enzymes provides a powerful strategy to identify possible targets for drug therapy. In this study we corroborated our previous results that suggested that *Dm* mtDNA helicase contains an ISC (Stiban et al., 2014), by demonstrating its presence in the NTD isolated from mitochondria of cultured *Drosophila* cells, and in a bacterial recombinant FL helicase. Furthermore, we determined that the ISC is important for the structural stability of the helicase, and can be substituted by Zn^2+^ ions. The ability to bind zinc ions is likely reminiscent of the viral origin of the mtDNA helicase, as an ortholog of bacteriophage T7 replicative helicase that coordinates Zn^2+^ (Kusakabe et al., 1999). The presence of the zinc ion in T7 gp4 was demonstrated empirically by the Richardson group using a chemical approach (Mendelman et al., 1994), which was verified by Ellenberger and colleagues using a structural approach (Kato et al., 2003). On the other hand, the replacement of Zn^2+^ with an ISC in the course of evolution suggests that the insect mtDNA helicase may have acquired a novel role, which given the general function of ISCs as electron carriers, may relate to fluctuations of redox conditions in mitochondria. ISCs, in contrast to lipids and DNA, are directly sensitive to reactive oxygen species (ROS) and hydrogen peroxide production (Scialo et al., 2013). Mitochondrial ROS mobilizes iron from ISCs (Marelja et al., 2018), and ISCs function as sensors of the mitochondrial redox environment. Mitochondrial complex I is the hub of ROS production in cells (Murphy, 2009).

The intracellular redox environment exerts effects on DNA binding activity and gene regulation of some DNA metabolizing enzymes (Choi et al., 2004). Replication of mtDNA is an energy- and resource-consuming process that may be downregulated in cells under conditions of excess ROS. Under oxidative stress, newly-synthesized DNA may represent a mutagenic target for superoxide anion-radicals. The presence of an ISC in *Drosophila* mtDNA helicase may render it vulnerable to excess ROS, allowing rapid removal of the cluster and a concomitant enzyme “switching off”. In this rheostat model, “switching off” *Dm* mtDNA helicase through its ISC would allow mitochondria to pause DNA replication until the environment is more favorable (a drop in ROS levels), or be turned over if the excessive ROS production persists. Though the human protein does not contain an ISC, a similar regulation may occur through other mechanisms such as oxidation of sensitive cysteine, tyrosine, lysine or arginine residues. Thus, it seems plausible that ROS signaling may control mtDNA replication in *Drosophila*, via the ISC in mtDNA helicase.

ISCs are typically transferred to their target proteins by dedicated ISC carrier proteins, and we have shown that *Dm* mtDNA helicase may interact functionally with *Dm* NUBPL. NUBPL proteins contain several conserved motifs, e.g., Walker A and B motifs, as well as the CXXC motifs that coordinate clusters (Via et al., 2000). *Dm* NUBPL exhibits their typical ATPase activity (data not shown) and dimeric structure. We showed that overexpression of *Dm* NUBPL in *Drosophila* cells reduces the characteristic pausing in mtDNA replication, supporting a role for ISCs in the replication process. Notably, high levels of *Dm* NUBPL alleviated pausing of replication at the slow zone 2, which is strikingly similar to the earlier reported effect of helicase overexpression (Ciesielski et al., 2018), suggesting a previous report of physical interaction between the two proteins (Guruharsha et al., 2011) also involves a functional interaction. The increase in the availability of ISCs by *Dm* NUBPL overexpression increases the efficiency of mtDNA replication, suggesting that the process is limited by ISC availability, which may in turn, have regulatory implications. Given that NUBPL orthologs are primarily responsible for the delivery of ISCs to complex I (Bych et al., 2008; Sheftel et al., 2009), it seems possible that *Dm* NUBPL also serves this function. If so, a shared dependence of mtDNA replication and the ETC on *Dm* NUBPL ISC transfer would suggest a reciprocal regulation, in which the levels of ISCs would promote mtDNA synthesis, and a subsequent increase in the abundance and/or the efficiency of ETC complexes that utilize them. Conversely, inhibition of mtDNA replication in the absence of sufficient levels of ISCs, would limit synthesis of new subunits of the ETC complexes. Chen and colleagues have proposed a model of selective inheritance through replication competition, in which there is direct link between functional respiring mitochondria and mtDNA replication (Chen et al., 2020). One might then speculate that ISCs serve as a link between oxidative phosphorylation and mtDNA replication in *Drosophila*.

The putative co-dependence of mtDNA replication and the ETC is supported by our finding that *Dm* mtDNA helicase and *Dm* NUBPL may co-associate with the MIM. *Dm* NUBPL bound liposomes tightly, independent of their composition. Binding was partially sensitive to increasing salt concentrations, suggesting that hydrophobic interactions may be contributory. This would support its expected role in providing ISCs to membrane-embedded proteins. Similarly, we showed that *Dm* mtDNA helicase (both the NTD and FL forms) associate with liposomes as did both the human FL mtDNA helicase and its RPD variant. Because the human RPD and FL proteins showed a nearly identical quenching pattern, it seems likely that the bulk of lipid binding occurs within the RPD; because the human mtDNA helicase lacks an ISC, the presence of a cofactor can not be a requirement for lipid binding. The association of human mtDNA helicase with the MIM was shown previously (Wang and Bogenhagen, 2006), and other mitochondrial helicases, such as Pif1p in yeast (Cheng and Ivessa, 2010) and human MDDX28 (Valgardsdottir et al., 2004) were also shown to do so. More recently, Spelbrink and colleagues demonstrated that endogenous human mtDNA helicase is attached firmly to the MIM even in the absence of mtDNA (Rajala et al., 2014). The association of mtDNA helicase with the MIM may enhance its ability to sense the redox environment because superoxide anion-radicals are formed near the matrix surface of the membrane, namely at FMN site of complex I or Qi site of complex III. We hypothesize that the NTD of mtDNA helicase is responsible for membrane binding, and show that the purified *Dm* NTD is a peripheral membrane protein that binds specifically to CL-rich membranes, which are similar to MIM composition rather than cholesterol-rich fractions.

In sum, current data support a model in which the mitochondrial nucleoid and its replication machinery are proximal to the MIM, and that the NTD in mtDNA helicase associates with the MIM via CL-enriched domains. In *Drosophila*, membrane binding and the concomitant transfer of an ISC from an associated *Dm* NUBPL may activate the helicase to initiate mtDNA unwinding and replication.

## Conclusion

The N-terminal domain of the *Dm* mtDNA helicase plays roles in binding cofactors, membranes and DNA. In particular, phospholipid membrane binding, which appears to be more specific for liposomes that mimic the lipid content of the MIM, supports the idea that the NTD of the helicase docks to the MIM, and recruits mtDNA to initiate the DNA unwinding process and replication in mitochondria. Considering the recent discovery of a DNA-CT role in the human nuclear, 4Fe-4S cluster-containing DNA primase (O’Brien et al., 2017), we propose that DNA binding by *Dm* mtDNA helicase is potentially regulated by redox signaling through the ISC in the NTD by DNA-CT.

## Data Availability

The raw data supporting the conclusion of this article will be made available by the authors, without undue reservation.

## Abbreviations

BODIPY-TMR-PI(4,5)P_2_, BODIPY® TMR Phosphatidylinositol 4,5-bisphosphate; CIA, cytosolic ISC assembly; CL, cardiolipin; *Dm*, *Drosophila melanogaster*; DNA-CT, DNA-mediated charge transfer; ETC, electron transport chain; FL, full-length; *Hs*, Homo sapiens; ISC, iron-sulfur cluster; mtDNA, mitochondrial DNA; MIM, mitochondrial inner membrane; Ni-NTA, nickel-nitrilotriacetic acid; NTD, N-terminal domain; NUBPL, nucleotide binding protein like; PS, phosphatidylserine; RPD, RNA polymerase domain; S2, Schneider 2; ROS, reactive oxygen species.
