# Supplementary figures and images for: Implications of Membrane Binding by the Fe-S Cluster-Containing N-Terminal Domain in the Drosophila Mitochondrial Replicative DNA Helicase
Source: Front Genet. 2021 Dec 7;12:790521. doi: 10.3389/fgene.2021.790521 (PMC8688847; doi:10.3389/fgene.2021.790521)

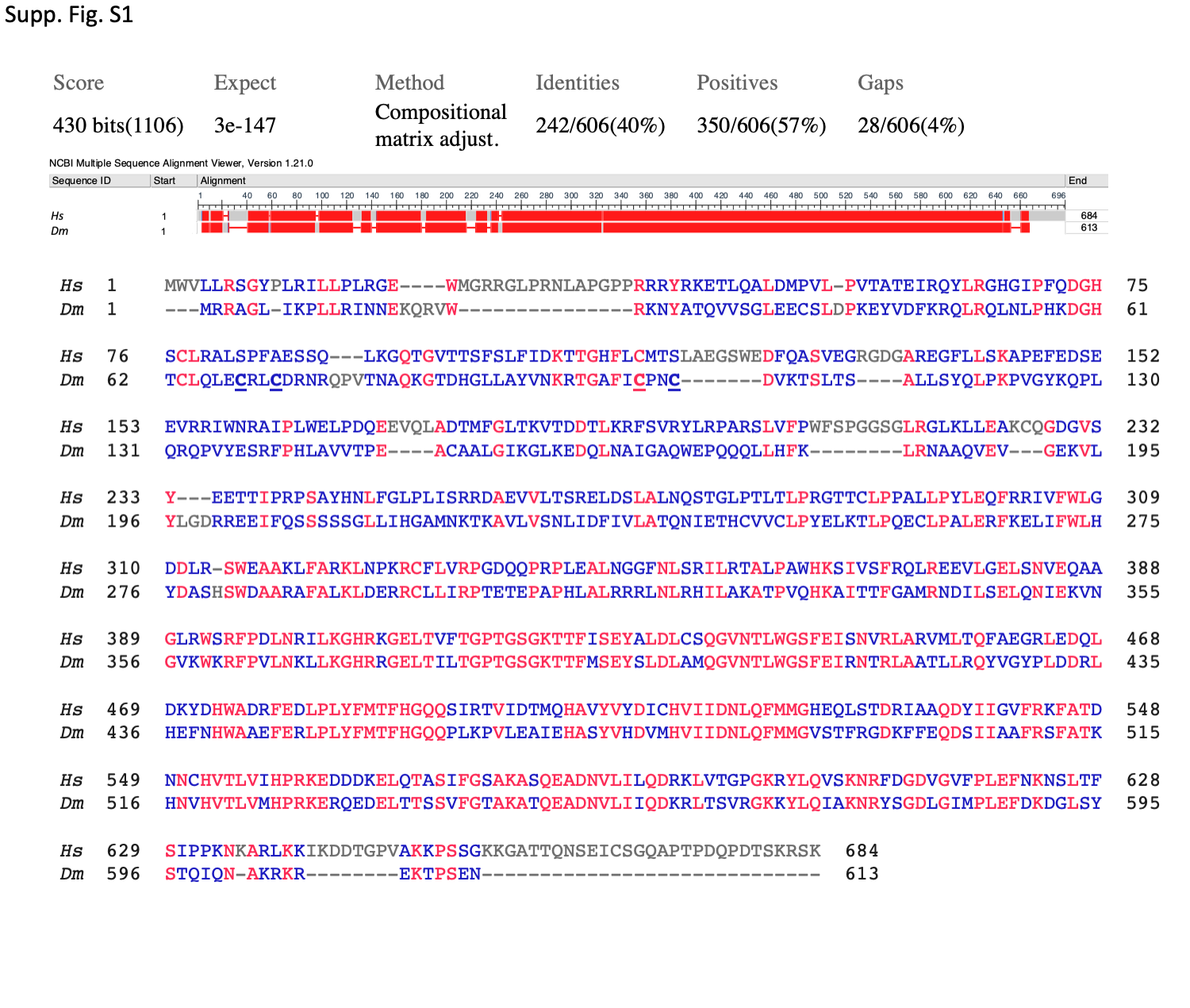

Supplement: Supplementary file 1 [file Image1.tiff]

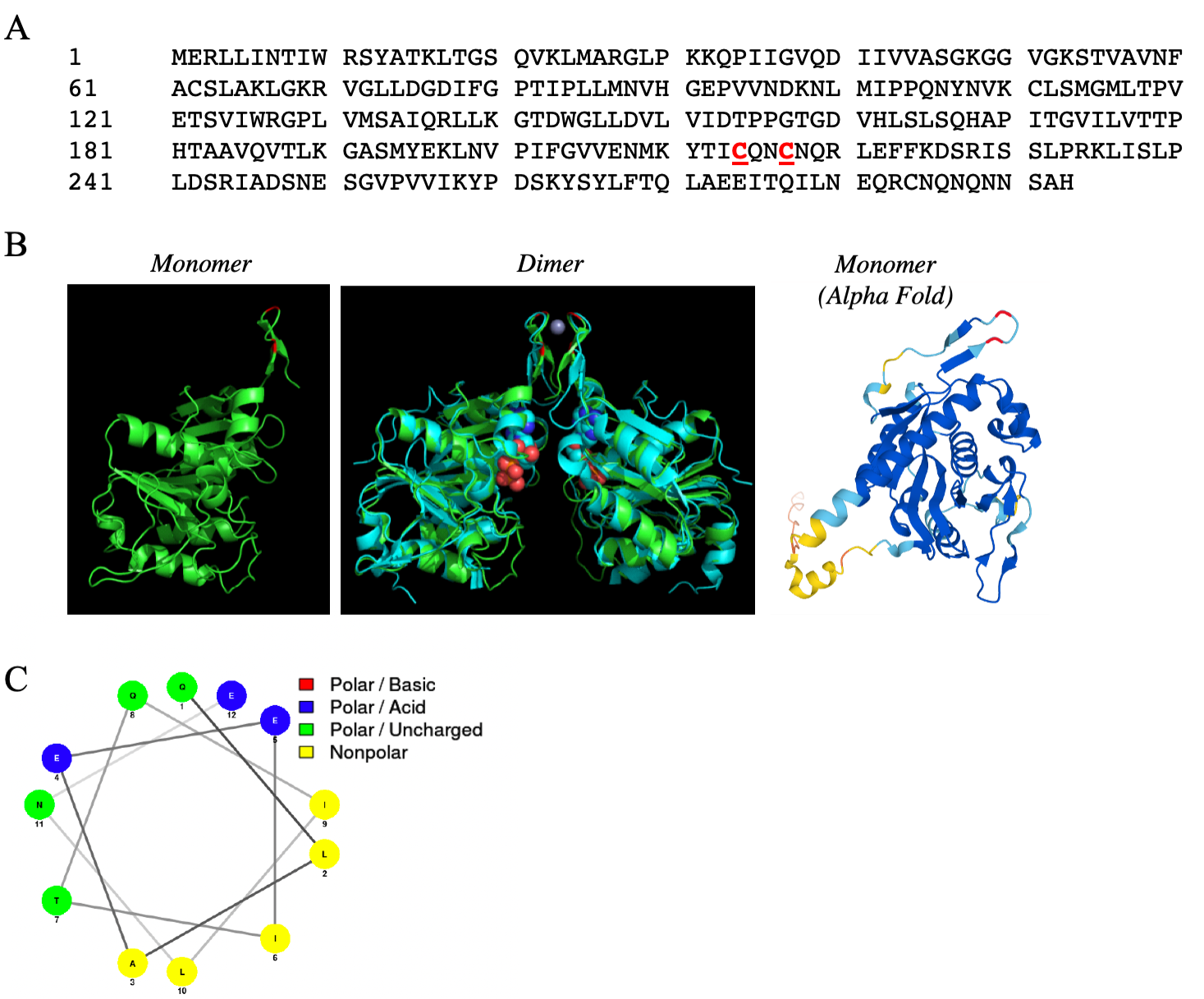

Supplement: Supplementary file 2 [file Image2.TIFF]
